# Who is on the primary care team? Professionals’ perceptions of the conceptualization of teams and the underlying factors: a mixed-methods study

**DOI:** 10.1186/s12875-017-0685-2

**Published:** 2017-12-28

**Authors:** Kirti D. Doekhie, Martina Buljac-Samardzic, Mathilde M. H. Strating, Jaap Paauwe

**Affiliations:** 1 Erasmus School of Health Policy and Management (ESHPM), Erasmus University Rotterdam, PO Box 1738, Rotterdam, 3000 DR The Netherlands; 2 Department of Applied Economics, Erasmus University Rotterdam , PO Box 1738, Rotterdam, 3000 DR The Netherlands; 3 Department of Human Resource Studies, Tilburg University, PO Box 90153, Tilburg, 5000 LE The Netherlands

**Keywords:** Primary care, Primary care professionals, Primary care teams, Teamwork, Team fluidity, Relational coordination, Mixed-methods

## Abstract

**Background:**

Due to the growing prevalence of elderly patients with multi-morbidity living at home, there is an increasing need for primary care professionals from different disciplinary backgrounds to collaborate as primary care teams. However, it is unclear how primary care professionals conceptualize teams and what underlying factors influence their perception of being part of a team. Our research question is: *What are primary care professionals’ perceptions of teams and team membership among primary care disciplines and what factors influence their perceptions?*

**Methods:**

We conducted a mixed-methods study in the Dutch primary care setting. First, a survey study of 152 professionals representing 12 primary care disciplines was conducted, focusing on their perceptions of which disciplines are part of the team and the degree of relational coordination between professionals from different disciplinary backgrounds. Subsequently, we conducted semi-structured interviews with 32 professionals representing 5 primary care disciplines to gain a deeper understanding of the underlying factors influencing their perceptions and the (mis)alignment between these perceptions.

**Results:**

Misalignments were found between perceptions regarding which disciplines are members of the team and the relational coordination between disciplines. For example, general practitioners were viewed as part of the team by helping assistants, (district) nurses, occupational therapists and geriatric specialized practice nurses, whereas the general practitioners themselves only considered geriatric specialized practice nurses to be part of their team. Professionals perceive multidisciplinary primary care teams as having multiple inner and outer layers. Three factors influence their perception of being part of a team and acting accordingly: a) knowing the people you work with, b) the necessity for knowledge exchange and c) sharing a holistic view of caregiving.

**Conclusion:**

Research and practice should take into account the misalignment between primary care professionals’ perceptions of primary care teams, as our study notes variations in the conceptualization of primary care teams. To enhance teamwork between professionals from different disciplinary backgrounds, professionals acknowledge the importance of three underlying conditions: team familiarity, regular and structured knowledge exchange between all professionals involved in the care process and realizing and believing in the added value for patients of working as a team.

**Electronic supplementary material:**

The online version of this article (10.1186/s12875-017-0685-2) contains supplementary material, which is available to authorized users.

## Background

The number of elderly patients aged 65 years or older with multiple chronic conditions living at home is rapidly rising [1, 2]. Research shows different high prevalence rates of elderly with multiple chronic conditions worldwide, ranging between 55% and 98% [3]. Because of their high and complex needs, care for these patients is delivered by multiple primary care professionals from different disciplinary backgrounds [1, 4]. Strong collaboration between these professionals is important [5], as it can lead to better patient outcomes in terms of patient-centred, high quality care and can improve not only patient satisfaction with care [6, 7], but also work satisfaction of professionals [8].

In research, the concept of collaboration is often used as a general term to describe a range of collaborative structures [9–12]. For example, a collaboration could consist of professionals with minimal interaction and no shared goal [10, 11]. Members of these type of collaborations are more task focused and often feel little necessity for interpersonal contact [10]. This type of collaboration is often defined as a ‘group’ or ‘network’ [10, 11]. A collaboration could also exist of members with a shared common goal, well defined tasks, task interdependency and stable membership [13]. Historically, this type of collaboration is defined as a ‘team’ [10, 11, 13].

In primary care, collaborations are often defined as ‘primary care teams’ [14–16]. Professionals from different disciplinary backgrounds can collaborate in formal structures, for example within the same organization, in which primary care teams are purposefully established, a common and shared care goal is set and professionals fulfil designated roles within the team. In such teams, accountability and procedural structures are embedded in the team, and the team membership of primary care professionals is clear [10, 17].

However, in recent years, teams have tended to become more fluidly structured, operating within loose boundaries and accordingly leading to dynamic team membership [7, 13, 17–21]. Present-day teams are expected to continuously and rapidly adapt to changes and issues in their environment, for example to changing patient expectations and demands [13, 22]. Team membership has a dynamic nature [13]; therefore, professionals can be members of multiple teams at the same time (i.e., multiple team membership) [13, 23]. Such teams are conceptualized as fluid entities in which membership is based more on task interdependency than formal structures [10, 13, 17]. Fluid teams are often described as having an ad hoc or multi-layered structure [13, 17]. In ad hoc teams, a team is built of members with diverse expertise to address specific needs, after which the team is dissolved and a new team is built [13, 20]. Teams with multi-layered structures consist of multiple inner and outer layers. The inner layer is formed by members who have a central and permanent role in the team, whereas the outer layers consist of team members who are members for a limited time period during which their specific expertise is required [13, 24].

Research on team fluidity shows both positive and negative effects of having dynamic team membership. By increasing the diversity of knowledge, team creativity and the opportunity for open discussions can be enhanced, which ultimately positively affects team performance [25, 26]. However, dynamic team membership can also lead to less coordination and team familiarity, as team members have less shared work experience [19, 21]. According to Mortensen [21], dynamic team membership can lead to a misalignment of team members’ perceptions regarding who is considered part of the team, which is referred to as the membership divergence phenomenon.

Although one could argue that the membership divergence phenomenon [21] may be an issue in the primary care setting due to the variety of conceptualizations of primary care teams, little research has focused on primary care professionals’ perceptions of team membership [27]. Therefore, the basic questions regarding how primary care professionals conceptualize teams and whether they perceive themselves as working as a team with professionals from other disciplinary backgrounds remain unanswered. Due to the changing and more fluid structure of present-day teams, is it debatable whether the term ‘team’ is still the appropriate term to describe these type of collaborations [17]. Regardless of the structures or perceptions of the type of collaborations between primary care professionals, collaborations are frequently labelled as teams merely on the assumption that teamwork will lead to superior outcomes [14, 15]. This phenomenon is discussed by Allen and Hecht [28] as the ‘romance of teams’. However, if primary care professionals do not perceive themselves as working as a team, it may not result in superior outcomes and is more likely to negatively affect their collaboration and ultimately the quality of care [10, 29].

Research suggests different underlying factors that could influence professionals’ perceptions of which disciplines they consider to be part of a team [8, 21–31]. These factors revolve around the presence of formal work processes within teams (e.g., communication, clearly defined goals and regular feedback loops to improve team performance [7, 8, 30, 32]) and informal social processes (e.g., mutual respect, trust and understanding of each other’s roles) [7, 29, 31, 32].

The interrelation between formal and social processes in teams is described in the ‘relational coordination’ theory [33, 34] and is defined as “*a mutually reinforcing process of interaction between communication and relationships carried out for the purpose of task integration*” [33]. This theory identifies key concepts regarding the communication and relationship ties between team members that underpin effective teamwork [34]. The quality of communication consists of four dimensions: frequency, timeliness, accuracy and a focus on problem solving rather than blaming [35]. The quality of relationships consists of three dimensions: the extent to which team members have shared goals, shared knowledge and mutual respect [35]. Although the relational coordination theory often focuses on the ties between core team members, Gittell [36] pleas for an extension of the theory beyond the inner layer and to include relational coordination with non-core participants (i.e., the outer layers), as these participants may also play an important role in the work process [13]. This approach emphasizes the importance of including a broad range of team structures and taking team fluidity into account.

Research on relational coordination suggests an interaction and mutually reinforcing effect between the degree of relational coordination and professionals’ perceptions of team membership. On the one hand, relational coordination can positively affect the perception of team membership [37]. Research shows that for primary care delivery, specifically disease-management programmes for chronically ill patients, higher degrees of relational coordination exist between professionals from different disciplinary backgrounds compared to professionals from the same disciplinary background [38]. This could be explained by the emphasis of disease-management programmes on multidisciplinary interactions, as the effectiveness of chronic care delivery is dependent on the communication and relationships between professionals [38]. Following this line of reasoning, we could say that professionals from different disciplinary backgrounds who share high degrees of relational coordination are more likely to perceive each other as members of the same team and to collaborate as a team. On the other hand, the degree of relational coordination between professionals could be enhanced by facilitating interactions between professionals in multidisciplinary meetings [39]. This suggests that when professionals from different disciplinary backgrounds get the opportunity to meet each other, they are more likely to perceive each other as part of the same team, which could result in higher degrees of relational coordination.

In this study, the perceptions of primary care professionals from different disciplinary backgrounds are our central focus. We aim to provide more insight into the concept of primary care teams and the functioning of these teams from the perspective of primary care professionals themselves. Our research question is the following: *What are primary care professionals’ perceptions of teams and team membership among primary care disciplines and what factors influence their perceptions?*

## Methods

In this paper, a sequential mixed-methods approach was used. First, a questionnaire survey study was conducted focusing on the perceived team membership and relational coordination between professionals from different backgrounds. The quantitative results showed a misalignment of the perceptions of professionals from different disciplinary backgrounds regarding which disciplines were part of the team. This analysis will be discussed in more depth in the results and discussion sections. Subsequently, semi-structured interviews with professionals representing different primary care disciplines were conducted to gain a deeper understanding of the misalignment and insight into the influencing factors.

### Setting and participants

This study was performed in the primary care setting in the Netherlands. The Netherlands, comparable to other European countries such as the United Kingdom and Denmark, has been identified as having a strong primary care system with high access to primary care [40, 41]. Similar to systems in Italy, Norway, Sweden and Estonia, primary care in the Netherlands is characterized by a referral system to secondary care and a gatekeeping position of general practitioners [41, 42]. Although different professionals are considered to be primary care professionals, such as physiotherapists and pharmacists, general practitioners are seen as the central care providers and first contact persons in care for patients [41]. These professionals deal with a large range of health problems and diseases and patients need to obtain their referral to medical specialist care. Moreover, practice nurses play a more central role in care in countries such as the Netherlands, Poland and Sweden [41]. In the Netherlands, these nurses often provide health programs such as dietary programs to elderly and sometimes focus on a specific patient groups like diabetic patients [41].

In light of the growing aging population, many European countries such as the Netherlands, France and Germany emphasize ‘ageing in place’: treating patients at home for as long as possible [43]. From this viewpoint, these countries have restructured their health system with a decentralization of government responsibilities at local (municipality) level, focusing on strengthening the primary care system [43, 44]. With this decentralization, patients need to live independently at home for as long as possible and rely on their informal care network before applying for professional care provision [43].

In this paper, we solely focus on the perceptions of primary care professionals and exclude social care and informal caregivers. Prior to the data collection for the questionnaire, the researchers composed a list of common primary care disciplines involved in care for chronically ill elderly patients based on existing research on primary care (Table 1) [45]. Convenience sampling was used to select participants. Managers of multiple types of primary care practices, for example primary care centres and monodisciplinary centres such as general practitioner centres, were approached by telephone or email.

**Table 1:** List of common primary care disciplines

1. General Practitioners	7. (District) Nurses
2. General Practitioner Assistants	8. Helping Assistants
3. Physiotherapists	9. Primary Care Psychologists
4. Remedial Therapists	10. Geriatric Specialized Practice Nurses
5. Pharmacists	11. Occupational Therapists
6. Dieticians	12. Speech Therapists

The questionnaires were filled out anonymously. Informed consent was assumed by completion of the questionnaire. Participants were given two weeks to complete the questionnaire. After one week, a reminder was sent to all participants. The total sample consisted of 152 primary care professionals from 37 different primary care organisations (response rate of 38%). The participant characteristics can be found in Table 2.

**Table 2:** Quantitative survey: Participants characteristics (*n* = 152)

Characteristic	*n*	%	
Sex			
Male	33	21.7	
Female	119	78.3	
Education level completed			
Secondary school	13	8.6	
Secondary vocational	35	23	
Bachelor degree	89	58.6	
Master degree	14	9.2	
Other	1	0.7	
Discipline			
Physiotherapist	36	23.7	
Helping Assistant	31	20.4	
Remedial Therapist	22	14.5	
(District) Nurse	19	12.5	
General Practitioner Assistant	12	7.9	
General Practitioner	9	5.9	
Primary Care Dermatologist	6	3.9	
Geriatric Specialized Practice Nurse	5	3.3	
Dietician	5	3.3	
Occupational Therapist	2	1.3	
Speech Therapist	2	1.3	
Primary Care Psychologist	2	1.3	
Other	1	0.7	
	Mean	SD	Range
Team tenure	6	7.2	1–35
Age (years)	40	12.1	21–64
Team size	9.9	5.4	2–40
Team diversity	.46	.30	0–.93
Note. SD = Standard deviation			

For the interviews, professionals from five main primary care disciplines were approached. In order to determine these five disciplines, we analysed our quantitative results and organized meetings with stakeholders in primary care. This approach resulted in the inclusion of the following disciplines: general practitioners, physiotherapists, occupational therapists and (district) nurses. During the interviews, multiple participants emphasized the importance of geriatric specialized practice nurses in elderly primary care. This discipline was therefore included as well. Convenience sampling and a snowball method were used to select participants. We conducted interviews until no new perspectives or underlying factors were being offered (i.e., saturation strategy), which finally resulted in 32 interviews. During the recruitment process of the participants as well as at the start of the interviews, all participants were repeatedly informed on the aims and purpose of the study. Informed consent was assumed by agreeing and completion of the interviews. Moreover, participants were repeatedly informed about the recording the interviews. At the start of each interview, the participants were explicitly asked for verbal consent for recording of the interviews. At all times, participants were allowed to withdraw their consent and end the interview. The participant characteristics can be found in Table 3.

**Table 3:** Qualitative interviews: Participants characteristics (*n* = 32)

Variable	General Practitioner (*n* = 6)	Physio- therapist (*n* = 7)	Occupational therapist (*n* = 7)	(District) Nurse (*n* = 9)	Geriatric specialized practice nurse (*n* = 3)
Gender					
Male	2	2	0	1	0
Female	4	5	7	8	3
Age in years					
< 30	0	2	2	3	0
30–50	5	3	4	2	2
> 50	1	2	1	4	1
Work setting					
Home care organization	0	0	0	9	0
General practitioner centre	2	0	0	0	2^a^
Physiotherapy centre	0	3	0	0	0
Occupational therapy centre	0	0	5	0	0
Primary health care centre	4	4	2	0	1
Number of years practicing					
< 15	2	3	3	5	1
15–30	1	2	3	1	2
> 30	3	2	1	3	0

^a^In the Netherlands, geriatric specialized practice nurses often work within general practitioner centres

### Quantitative questionnaire

The questionnaire was divided into two sections. The first section contained two questions on primary care professionals’ perceptions regarding team membership, focusing on their perceived team size (“How many team members are on your team?”) and team diversity (“Which of the following disciplines do you consider part of your team?”). The latter question was based on Table 1. Participants were asked to answer openly, without reference to their specific work setting or structure. For example, the general practitioners were asked to indicate which other primary care disciplines (as presented in Table 1) they considered part of their team. In the second section, the degree of relational coordination was assessed using a seven-item relational coordination scale. This scale was originally developed to measure airline operations [46] but has also been applied in health care settings [34]. Sample questions include “How frequently do you communicate with each of these disciplines about a patient?” and “To what degree do people in these disciplines share your goals for the care of your patients?” A five-point Likert scale ranging from 1 (“never”) to 5 (“always”) was used. Participants were asked to answer the questions with respect to the other disciplines. For example, the physiotherapists were asked to score how frequently they communicated with the helping assistants about a patient. The used questionnaire can be found in Additional file 1. Principal component analyses revealed that the seven items loaded onto two factors with eigenvalues of 3.53 and 1.48, which explained 71.61% of the variance.

### Qualitative interviews

The topic list for the semi-structured interviews was developed by the primary researcher and revised based on input from the full research team. The design of the topic list allowed an in-depth investigation of the underlying dimensions of the misalignment on team membership among professionals. Participants were first asked how they would define ‘teams’ and if they felt to be members of a team. Example questions include “What elements of teamwork could make you feel more like a member of a team?” and “What is important to you when collaborating with other disciplines?” The interview guide can be found in Additional file 2.

### Quantitative analysis

The data were analysed using IBM SPSS 22.0. Descriptive statistics were used to analyse the sample characteristics, the perceived team size, and the relational coordination between disciplines. Each discipline’s perceived team diversity was analysed using Blau’s index for diversity [47]. The index ranges between zero (completely homogeneous teams) and one (completely heterogeneous teams). To explore the different perceptions among disciplines regarding who is a member of the team, UCINET Software for Social Network Analysis was used to create a social network figure of the different disciplines. To analyse the relationship between perceptions regarding who is part of the team and the degree of relational coordination between disciplines, correlation analysis was performed.

### Qualitative analysis

The interviews were audiotaped, transcribed verbatim and analysed using Atlas TI (version 7). Data analysis was a combination of inductive coding and deductive framework analysis and included several steps. First, the primary researcher read the transcripts multiple times to gain a preliminary understanding of the experiences of the participants. Then, the primary researcher initiated an open coding of all the data. Next, the full research team compared the codes to derived insights from literature on teams and team membership. Specific attention was paid to participants’ conceptualization of primary care teams and factors that participants mentioned that could increase their perception of being part of a primary care team. During this process, the codes found from the open coding process were grouped into sub-themes, which were then grouped into major themes. For example, the codes ‘flying in and out’ and ‘loose boundaries’ were grouped into the subtheme ‘team versus loose network’, which was then included under the major theme ‘conceptualization of teams’. To ensure reliability, the themes were discussed among the full research team until consensus was reached, which was the case after five meetings with the full research team.

## Results

### Quantitative results

#### Who is part of the team

The average indicated team size was 9.9 members and the average diversity in disciplines in the team was .46. When specifying team size per discipline (Additional file 3), primary care dermatologists reported the largest team size (15 members), and primary care psychologists reported the smallest team size (7 members). Regarding team diversity, occupational therapists reported the highest diversity (.79), and remedial therapists reported the lowest diversity (.09).

**Fig. 1:**
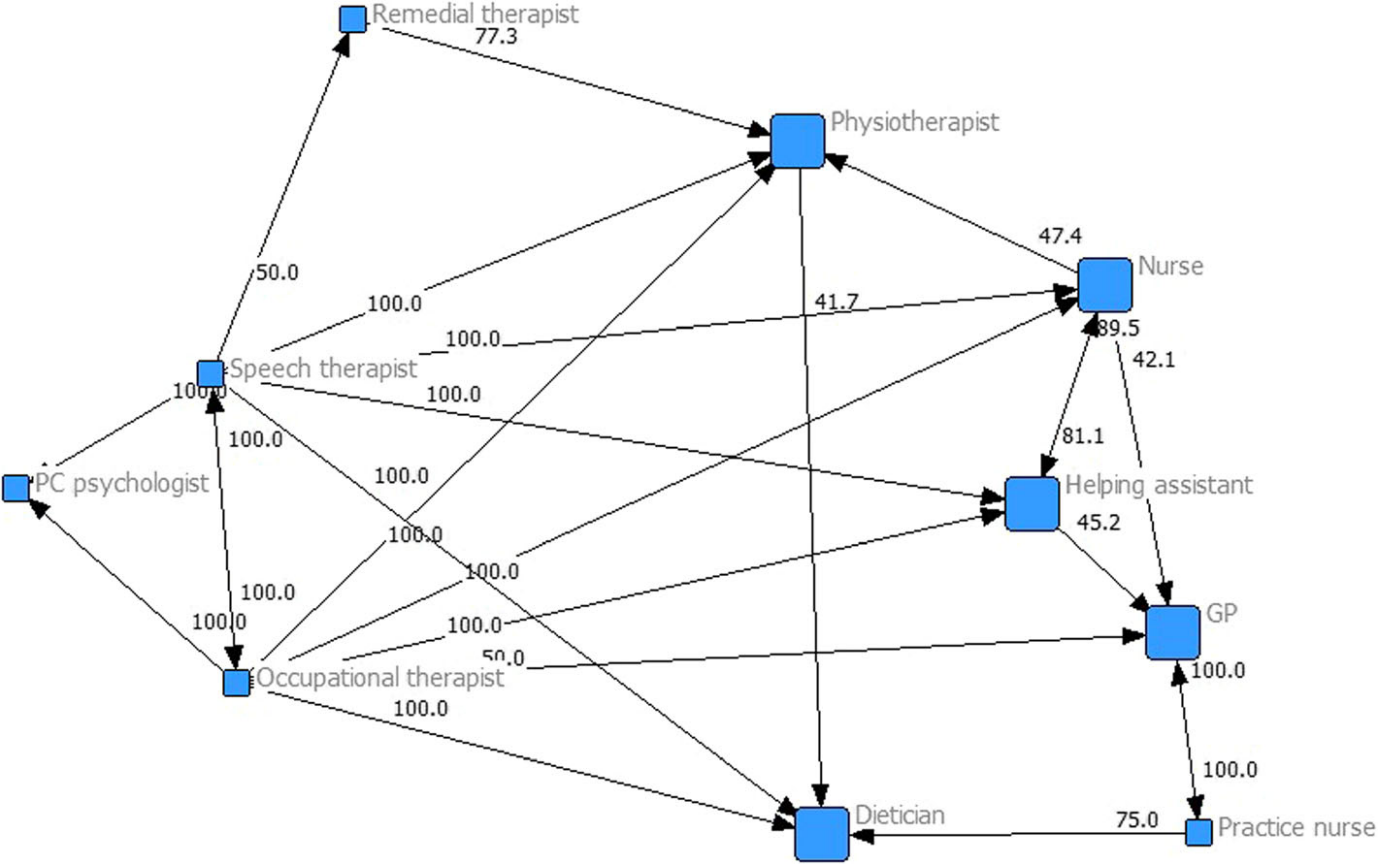
Social network figure on professionals’ perceptions on team membership. The social network figure illustrates participants’ perceptions of which disciplines are part of their team. The arrows and percentages show which disciplines and how many participants from a specific disciplinary background consider another discipline to be part of the team. The arrows represent percentages higher than 40%. The absence of an arrow implies a percentage lower than 40%. All percentages, also lower than 40%, are provided in Additional file 3. For example, Fig. 1 shows that 77.3% of the remedial therapists considered a physiotherapist to be part of the team

Alignments and misalignments between the perceptions of professionals from different disciplinary backgrounds were found, as illustrated in Fig. 1. Notably, most arrows point towards physiotherapists, general practitioners, dieticians, helping assistants and (district) nurses, which indicates that these disciplines were most often considered to be part of the team. Helping assistants and (district) nurses consider each other to be part of their team; 89.5% of the helping assistants consider a (district) nurse to be part of their team and 81.1% vice versa. There is a maximum alignment of perceptions between general practitioners and geriatric specialized practice nurses, as both disciplines considered each other to be part of their team at a level of 100%. Although general practitioners only considered geriatric specialized practice nurses to be part of their team, they were considered part of the team by three additional disciplines: helping assistants (45.2%), (district) nurses (42.1%) and occupational therapists (50%), indicating a misalignment in perceptions between general practitioners and these disciplines. Physiotherapists were considered to be part of the team by the remedial therapists (77.3%), speech therapists (100%), occupational therapists (100%) and (district) nurses (47.4%), whereas less than 40% of the physiotherapists considered any of these disciplines to be part of their team. Moreover, no arrows are present between physiotherapists and general practitioners or between general practitioners and dieticians, indicating that less than 40% of these disciplines considered one another to be part of their team.

#### Relational coordination

A (mis)alignment of perceptions was also found with regard to the perceived degrees of relational coordination. On a scale of one to five, Additional file 4 shows the average degree of relational coordination between professionals from different disciplinary backgrounds, subdivided into the communication and relationship dimensions. Overall, the means scores on the communication dimensions are lower than those on the relationship dimensions.

Helping assistants and (district) nurses not only often perceive each other’s discipline to be part of the team (Fig. 1), but also share roughly similar degrees of relational coordination on both dimensions. From the perspective of (district) nurses, the perceived degree of relational coordination on the communication dimensions with helping assistants was 4.54, and the perceived degree of relational coordination on the relationship dimensions was 4.49. Vice versa, the perceived degree of relational coordination on the communication dimensions was 4.74 and on the relationship dimensions 4.78. Misalignments in the degrees of relational coordination were found for general practitioners. For example, from the perspective of (district) nurses, the perceived degree of relational coordination with general practitioners on the communication dimensions was 3.43 and on the relationship dimensions 3.64, while vice versa, the perceived degrees of relational coordination were 2.44 and 2.48, respectively.

Additional file 5 shows the correlation matrix between a specific discipline perceived to be part of the team and the perceived degree of relational coordination with that discipline, subdivided into the communication and relationship dimensions of the relational coordination theory. Overall, higher correlations were found between being perceived as part of the team and the communication dimensions of relational coordination (i.e., frequency, timeliness, accuracy and a focus on problem solving rather than blaming), but the overall mean scores for the relationship dimensions were higher. Following the identified misalignment in perceptions as illustrated in Fig. 1 and Additional file 4, the correlation matrix shows ambiguous relationships between a specific discipline considered to be part of the team and the perceived degree of relational coordination (i.e., communication and relationship) with that discipline. For general practitioners, there are relatively low correlations between perceiving that discipline to be part of the team and both the communication dimensions and the relationship dimensions (*r* = .35 and *r =* .17 respectively), although 40.9% of the participants considered general practitioners to be part of their team. Conversely, high correlations were found for primary care psychologists on both the communication dimensions and the relationship dimensions (*r* = .65 and *r* = .56 respectively), although only 18.8% of the participants considered this discipline to be part of their team. The (district) nurses and helping assistants showed high correlations on both the communication (*r* = .76 and *r* = .67 respectively) and relationship (*r* = .76 and *r* = .68 respectively) dimensions.

#### Qualitative results

The aim of the interviews was to investigate the reasoning for the identified misalignment of the perceptions of primary care professionals regarding which other disciplines they consider to be part of their team. Analyses of these interviews showed two lines of reasoning. The first theme, ‘conceptualization of teams’, focuses on the different perspectives of the participants regarding the concept of primary care teams. Second, our analysis identified three factors that could minimize the misalignment of perceptions: ‘knowing the people you work with’, ‘the necessity for knowledge exchange’ and ‘sharing a holistic perspective of caregiving’. These three factors are combined in the theme ‘factors influencing the perception of working as a team’. Specific quotes are included under each (sub) theme to provide meaning and context to the participants’ perspectives.

#### Conceptualization of teams

The first theme involved the meaning of the concept of teams. Most participants mentioned that teams consist of multiple layers. According to most of the general practitioners, the inner layer is formed by the general practitioners themselves, geriatric specialized practice nurses and (district) nurses, as these three disciplines are considered to have central tasks in caregiving and are involved for a long period of time. This contrasts the questionnaire results, in which less than 40% of the general practitioners considered the (district) nurse part of the team. The outer circles are formed by professionals whose expertise is needed for a limited period of time. According to the general practitioners, these professionals are often physiotherapists and occupational therapists.

“*A team to me is when together you provide high quality care for a patient. A network is more like loose grains of sand. A real team is often the general practitioners, the home care organization and the practice nurses. And occasionally, other people [KD: disciplines] are flown in like a physiotherapist or an occupational therapist. But the core of the team really is the general practitioner and the home care organization.” (General practitioner 1)*

“*For example, an occupational therapist can arrange walkers for patients with Parkinson’s. But long-term care, they don’t provide that. They are more or less flown in, do their business and fly out again. And it could very well be that you need them again later, but not structurally.” (General practitioner 2)*

However, most occupational therapists and physiotherapists felt that in the eyes of patients, they do belong to the core of the team around a patient, as their fields of expertise focus more on helping a patient with daily activities than treating their medical condition. According to the occupational therapists, knowing how to manage daily life and how to remain independent are important goals for patients.

Which disciplines are considered by a professional to be part of the inner layer and the outer layer seem to be related to the extent to which professionals from different disciplinary backgrounds are familiar with each other and the frequency of contact. Some participants felt a lower “team familiarity” with professional who they do not meet or speak to on a regular basis. The importance of knowing the people you work with in relation to perceiving to work as a team is described more in depth in the second theme.

“*Well, a social worker might be involved whom I have never spoken to or whose patient goals I might not know. That person will have a lower team familiarity towards me than the physiotherapist whom I regularly speak with regarding a client’s condition. That may be via phone or email, that’s not important to me. So in that sense there are multiple layers.” (Occupational therapist 1)*

The extent to which participants felt that they were part of a team was divided and seemed to be related to the type of work structure (i.e., working within the same building or not). Professionals working within the same building often referred to each other as members of the same team. However, for professionals who work in a monodisciplinary centre, the team concept applies to professionals from the same disciplinary background.

*“The centre I am currently working in does feel like a team, but actually, my team members are merely my fellow general practitioners.” (General practitioner 3)*

With regard to teamwork with professionals from other disciplinary backgrounds outside a formal structure or the same building, participants did not perceive to work as a team. These multidisciplinary collaborations were often described as “loose networks” around a single patient. The participants did not refer to these collaborations as teams because of the perceived incidental structure of the collaboration. Professionals who do not structurally work together for the same patient group are not perceived as a team.

*“It [KD: collaboration with different disciplines] doesn’t feel like a team because it’s usually a one-time collaboration around a patient. And perhaps you meet the same people around another patient, but that doesn’t make it a team. It’s more an incidental collaboration around a patient. So it’s more like a network.” (Occupational therapist 4)*

Although most participants felt that all professionals ultimately want the best care for their patients, the participants felt that professionals work individually with few mutual connections.

*“When I look at the care for the elderly that we give, I feel that the older person is at the centre and we as professionals stand around the patient. And everybody does their own thing. But it would be very nice if all of those professionals had connections with each other.” (General practitioner 5)*

### Factors influencing the perception of working as team

The three most mentioned factors that could influence the perception of working as a team are described below.

#### Knowing the people you work with

Having met the other professionals in person and knowing who that person is could positively influence communication and coordination by increasing the levels of familiarity and trust. Professionals know what to expect from each other, know their mutual responsibilities and can hold each other accountable for their actions. The occupational therapists, physiotherapists and geriatric specialized practice nurses particularly emphasized the importance of knowing the other professionals involved in the care for the same patient.

*“Well, there’s a difference between knowing each other in the sense of ‘I know the other person’s name’ and knowing in the sense of ‘I’ve seen his or her face’. If you recognize each other’s faces, the collaboration will be ten times better because usually right after five minutes you’ll know things like, ‘Oh, everything will be all right with that physiotherapist’, or ‘Oh, that general practitioner is very involved’.” (Occupational therapist 2)*

*“It does help a lot if you know each other. For example, the geriatric specialized nurse doesn’t work in this building, but since you know each other, you’ve already seen each other, and together you’ve invested time in knowing each other’s roles and expertise. You know what you can and can’t expect from each other. Or you can sometimes think along with another professional. That works really well, and I also think it’s important in elderly care.” (Physiotherapist 1)*

The general practitioners also acknowledged the positive effects of knowing the other professionals, but mentioned a lack of time as a hindering factor. Additionally, the fact that multiple professionals represent the same discipline in care for the same patient was viewed as a barrier to getting to know each other. This was especially the case for (district) nurses working in the same home care organization, where multiple (district) nurses can be involved in the care for a single patient.

Knowing each other could not only benefit the professionals but also the patients. Participants felt that patient satisfaction and patients’ trust in the care delivery could be increased if all of the professionals involved know each other and collaborate.

“*What I notice with the elderly people whom I visit is that they like it very much when everyone involved in their care knows each other. For example, when I visit a patient, and they say “Yeah, my physiotherapist is M!”, then I would say “Oh, I know her. I just saw her at another patient’s home”. “Oh that’s great!” So you can see that they like it when they know that you know the other professionals.” (Occupational therapist 6)*

THE NECESSITY FOR KNOWLEDGE EXCHANGE

Some participants expressed their desire for regular multidisciplinary team meetings to discuss patient cases. However, a lack of time often hinders the organization of these meetings. Communication therefore usually takes place via email or phone.

Both the frequency and the content of communication seem to be related to the degree of (task) interdependency between professionals and the patient’s medical condition. Regarding the frequency of communication, all participants acknowledged that communication mostly occurs when the coordination of tasks is necessary regarding a patient’s condition. When a patient is stable, communication is considered to be less necessary and thus less present. In that sense, communication is considered to be more incidental than structural.

*“I think that everyone [KD: primary care professionals] is highly involved in the care for patients with multi-morbidity, so there’s no real necessity to have contact in any way. Look, as far as I’m concerned, when things go really wrong, then there’s a need to deliberate.” (General practitioner 1)*

As a side note, compared to the other disciplines, occupational therapists and physiotherapists found it more important to update the other disciplines on their tasks on a weekly basis, especially the general practitioners. These disciplines found it particularly important to keep the other disciplines informed, as they highly valued providing holistic care to patients. However, communication is often felt to be one-directional; the general practitioners rarely respond to their emails.

Regarding the content of communication, participants found that information sharing solely focuses on a patient’s medical condition; the professionals rarely communicate for personal (social) reasons. Some participants, particularly the occupational therapists and physiotherapists, expressed a desire for more proactive communication between disciplines to prevent further deterioration of a patient’s condition. Instead of solely reacting to a patient’s – deteriorating – condition, professionals should more proactively communicate with other disciplines when their expertise could be helpful. Some occupational therapists expressed a desire for professionals to focus on multidisciplinary patient goals and not solely focusing on patient goals within their own field of expertise.

*“To me, it’s important that other professionals know how to find me if they have any questions regarding my treatment of a patient. For example, that they inform me when they see a patient goal related to occupational therapy. And that they share important developments in their own fields of expertise with me. I currently feel that I share what I am doing more often, that as an occupational therapist, I see patient goals within the field of expertise of other disciplines and make these disciplines aware of these goals than the other way around. That happens sometimes.” (Occupational therapist 4)*

SHARING A HOLISTIC VIEW OF CAREGIVING

Most participants felt that one of the core steps in enhancing teamwork is that each professional should have a holistic view of caregiving, meaning that professionals should not work individually and solely focus on the patient’s needs within their own field of expertise, but should collectively try to address all of the patient’s needs. Professionals should truly believe in the added value of working as a team around a patient instead of as distant individual professionals. As a result, they would actually want to work as a team. However, when professionals share little task interdependency, it can be difficult to see the added value of collaborating as a team, and they are therefore less likely to invest in teamwork.

*“It really also depends on your own perspective, whether you see each other as complementary and see each other’s added value, or if you rather like to keep things to yourself.” (Physiotherapist 7)*

Some (district) nurses expressed a desire for more teamwork with general practitioners, but they felt that the general practitioners often prefer to work solo. The results from the general practitioners on this matter were mixed. Some expressed a wish for more teamwork between different disciplines on a regular and structured basis, while others felt that teamwork is only necessary on an incidental basis when a patient’s condition is unstable.

*“General practitioners always say “we are so busy”. Nobody else in the world is busy, but they are. If we work with a general practitioner, he visits a patient on his own time. He doesn’t adapt to my schedule. It doesn’t matter if I’m there or not. It makes me sad because sometimes the patient needs a bandage and he [KD: general practitioner] won’t do it. We [KD: (district) nurses] are like a necessary evil. Nothing comes from the general practitioners that says that they’re willing to collaborate. The love always needs to come from the other side.” ((District) nurse 2)*

## Discussion

Our study explored the perceptions of primary care professionals from different disciplinary backgrounds regarding the conceptualization of teams and which disciplines they consider to be part of their team.

### Conceptualization of teams

Building further on the membership model divergence phenomenon of Mortensen [21], our study first shows that for the complex primary care setting professionals from multiple disciplinary backgrounds have different perceptions of which disciplines are part of a primary care team. For example, (district) nurses frequently consider general practitioners to be part of the team, but the latter often do not consider (district) nurses to be part of their team.

This misalignment can be linked to how primary care professionals conceptualize teams. In line with the team fluidity literature [13, 17], our study shows that primary care teams are perceived to have a fluid nature and consist of multiple inner and outer layers. Primary care teams have an inner layer consisting of disciplines with long-term involvement in care and outer layers of disciplines who are only team members when necessary. However, primary care professionals perceive which disciplines are part of the inner or outer layers differently. To illustrate, our interview results show that general practitioners do not consider occupational therapists as part of their team, because they help patients with specific problems and are only involved for a limited amount of time. However, the occupational therapists felt that they are part of the inner layer, giving their field of expertise to help patients with daily life activities.

Task interdependency is frequently mentioned as a core characteristic of teams [14, 48–50] and has been shown to positively affect team processes and effectiveness [8]. This study emphasizes the importance of task interdependency in primary care teams and suggests that the extent to which professionals perceive other disciplines to be part of the inner or outer layers of the team is dependent on task interdependency. When task interdependency is low, the perceived need for professionals to communicate and interact with other professionals in order to achieve their goals is also low. Consequently, these professionals are more likely to consider each other as members of the outer layer of a team. Vice versa, when task interdependency is high, professionals are more likely to consider each other part of the inner layer of the team, and the perceived need for communication and knowledge exchange will be higher.

In addition, this study emphasize the importance of the perceived goal interdependency, which refers to the interconnection among team members implied by the type of goal (individual or team) that guides their performance [51]. Professionals who perceive patient care as a holistic process and acknowledge that achieving patient goals from their own discipline is dependent on patient goals from other disciplines, will be more likely to want to collaborate as a team and consider each other as team members.

#### Underlying factors

For certain combinations of professionals from different disciplinary backgrounds, the (mis)alignment of perceptions regarding which disciplines are part of the team seems to be related to the perceived degree of relational coordination. For example, (district) nurses and helping assistants not only frequently consider each other to be part of the team but also share high degrees of relational coordination. This result suggests that (district) nurses and helping assistants often perceive each other to be part of the inner layer of a team and likely share equal expectations regarding, for example, their roles and responsibilities, shared goals and the frequency of their communication. When focusing on general practitioners in relation to the other disciplines, the expectations do not always align. For example, general practitioners are perceived to be part of the team by both (district) nurses and helping assistants, who also perceive relatively high degrees of relational coordination with general practitioners on both dimensions. However, vice versa, only a small percentage of the general practitioners consider these two disciplines to be part of the team, and their perceived degrees of relational coordination with (district) nurses and helping assistants are relatively low.

In practice, general practitioners are often considered to have a central role in the caregiving process [52]. Our study suggests that most primary care professionals acknowledge this central role of general practitioners, but that general practitioners do not always acknowledge the central role that other disciplines could play in the caregiving process. By having a highly medicalised focus on patient needs, some general practitioners tend to not perceive disciplines with a less medicalised contribution to patient care as part of the inner layer. However, these disciplines (e.g. occupational therapists) are crucial for patients’ quality of life as they focus on daily life activities. Our study also indicates a lack of or little communication between general practitioners and other disciplines. Communication was often a one way road towards general practitioners as they fail to respond to emails or phone calls. The misalignment in perceptions and lack of communication between general practitioners and other disciplines also suggests power differentials between the former and latter. Research shows that power can negatively affect team effectiveness [8] and affects the strategic choices of care professionals whether to collaborate, with whom and to what level [53]. Research by Rieck [54] on the relationship between general practitioners and pharmacists shows that power distances exist between these disciplines and is based on knowledge and expertise differences. General practitioners had little trust in the expertise of the pharmacists and felt to perform tasks better independently than as a team with the pharmacists. Following this line of reasoning, we could say that in our study, power differentials between general practitioners and other disciplines exist. General practitioners felt little necessity to function as a team with especially disciplines with a less medicalised contribution to care. This lack of communication and teamwork could negatively influence the quality of delivered care [55].

Our study suggests three underlying factors of the misalignment in perceptions: 1) knowing the people you work with, 2) the necessity of knowledge exchange, and 3) sharing a holistic view of caregiving. These factors are related to the communication and relationships between primary care professionals and could contribute to enhancing their perception of being part of a team.

In line with other studies such as that of Gucciardi and colleagues [32], our study emphasizes the importance of investing in communication and relationships between all professionals involved in the care for a single patient. Research has shown that high levels of trust, mutual respect and mutual understanding of each other’s roles are important characteristics of effective teamwork [7, 29, 32]. Building further on other research [32], the responses of our participants suggest that familiarity could increase mutual levels of trust, respect and understanding between professionals. By enabling primary care professionals from different disciplinary backgrounds to meet and get to know each other, trust, respect and understanding are nurtured. Furthermore, this study suggests that getting to know each other could also positively affect the quality of communication between professionals, as the professionals will be more familiar with each other.

#### Practice and research implications

To provide patient-centred holistic care to chronically ill elderly patients, all of the primary care professionals involved need to work together as one team, regardless of whether they perceive task interdependency. The importance of holistic care has often been emphasized in health policy, and our study shows that primary care professionals themselves acknowledge the need for a holistic view of caregiving. To provide holistic care, our study underscores the importance of strengthening the communication and relationships between professionals involved in the care for the same patient, as the professionals may have different expectations of each other.

In line with other research [32, 56], we suggest that to improve team functioning, all professionals involved in the care for the same patient could benefit from meetings in which they have the opportunity to get to know each other and discuss their mutual roles, responsibilities and expectations. Research stresses the importance of informal contact between team members to enhance role clarification and social processes within the team [30, 32, 57]. By organizing these meetings, professionals can build on their mutual levels of trust, respect and understanding, and role conflict can be minimized. However, it is crucial that hindering factors such as a lack of time, motivation and the perceived added value of informal contact and engaging with each other are taken into account. This could for example be realised by not organizing specific meetings focused on informal contact, role clarification and engagement, but to integrate these elements into multidisciplinary team meetings in which critical incidents are discussed. For example, informal contact, role clarification and engagement could be integrated into simulation-based trainings at the workplace for professionals who already work together or need to communicate and coordinate their activities [58]. Within these simulation trainings, professionals have the opportunity to re-enact a real life case to stimulate and improve their teamwork around a patient [58]. Research has shown that simulation training improves technical skills as well as non-technical skills of professionals [59–61]. Further research is needed to gain more knowledge of underlying conditions that are necessary for these meetings to succeed.

Moreover, different researchers [14–16, 28] have debated the definition of teams and have shown that the label “team” is often applied to a collaboration in the belief that teamwork leads to superior outcomes. Some research argues that in reality, many of these so-called teams consist of professionals who work individually, rarely communicate or do not share a common goal [14, 15]. Adding to this debate, this study shows that although a collaboration is frequently labelled as a primary care team, professionals from different disciplinary backgrounds often do not perceive themselves as part of a team and have different perceptions regarding which disciplines are part of the team. Rather, some primary care professionals may perceive that they work on a team, while others may perceive that they work in a network. However, as our study shows that these networks are multi-layered, the actual structure and formal and social processes within networks may vary in different contexts. This also implies that researched primary care teams or primary care networks may vary in their actual structure and membership, making it difficult for example to compare primary care team effectiveness across studies. Thus, policy-makers, managers and researchers should carefully consider the specific context in which teamwork takes place and the perceptions of professionals on team membership and the conceptualization of teams and networks. Our study shows that professionals may have different perceptions on team membership and task interdependency, frequency and content of communication between professionals varies. Therefore, when using the terms ‘primary care team’ or ‘primary care network’, it is important to specify who the members are and what their task interdependency and communication is.

Our study shows a misalignment in perceptions of primary care professionals and suggests different underlying factors influencing their perception. Further research is needed to more in depth explore this misalignment, for example by focusing on factors on a patient level. The extent to which professionals see each other as part of the inner or outer layer could be influenced by patient characteristics, such as complexity of patient condition, intensity of treatment and patient involvement.

Moreover, research on self-management and health care consultations underscores the importance of patient involvement and indicates that patients fulfil different roles, from passive recipients of care to active participants or co-producers of their care [62]. Therefore, future research on primary care teams should focus on the different roles of patients in the self-management of their diseases and on patients’ team membership in primary care teams. In addition, because of the growing prevalence of informal caregivers and their unique role as semi-patients and semi-professionals [63], future research should also focus on informal caregivers’ team membership.

#### Limitations

When interpreting our results, careful consideration must be paid to the following. First, due to our cross-sectional design, we cannot draw any conclusions regarding causality between the extent to which professionals perceive specific disciplines as part of their team and their perceived degree of relational coordination. At the same time, due to our mixed-methods design, our study does suggest that these concepts are related and that investing in communication and relationships between professionals is important for teamwork. Second, the quantitative component of our study included a moderate sample size and had an unequal distribution of participants per discipline. However, our sample included a large variety of primary care professionals from different disciplinary backgrounds, reflecting the diversity of primary care services for chronically ill elderly patients.

Third, in both the quantitative and well as the qualitative component of our study we did not include all primary care disciplines from the same primary care practice, such as all members of one community care team, because we aimed to openly explore the relationships between professionals from different disciplinary backgrounds. We therefore cannot draw any conclusions on (mis)alignments of perceptions between professionals involved in the care for a specific patient. Future research could focus on exploring teams and networks around a specific patient and the perceptions of the professionals within these structures.

## Conclusion

Our study shows that from the perspective of primary care professionals, the concept of primary care teams is ambiguous and misalignments exist regarding how these teams are conceptualized and which disciplines are perceived as part of the team. To create more alignment and to enhance professionals’ perceptions of being part of a team, professionals emphasize the importance of knowing the people you work with, exchanging knowledge with all professionals involved and sharing a holistic view of caregiving. By focusing on these underlying conditions of teamwork, professionals are not only more likely to perceive themselves and professionals from other disciplines as team members but are also more likely to collaborate as a team.

## Additional files

Additional file 1: Questionnaire survey data. The questionnaire used for quantitative data collection on collaboration and relational coordination between primary care professionals. (DOCX 59 kb)

Additional file 2: Interview guide. The interview guide used for qualitative data collection on collaboration and teams. (DOCX 16 kb)

Additional file 3: Team size, diversity and part of the team. The table indicates the average team size and diversity and which primary care disciplines are considered as part of the team by professionals from other disciplinary backgrounds. (DOCX 14 kb)

Additional file 4: Mutual degrees of relational coordination, subdivided into communication and relationship dimensions. The table indicates the degrees of relational coordination between primary care professionals from different disciplinary backgrounds as perceived by the professionals themselves. (DOCX 14 kb)

Additional file 5: Descriptives and correlations between perceived as part of the team and the degree of relational coordination. The table indicates the correlation between the perceptions of all participants on the team membership of professionals from a specific disciplinary background in the team and the perceived degrees of relational coordination with these professionals. (DOCX 13 kb)
