# Supplementary material for: Who is on the primary care team? Professionals’ perceptions of the conceptualization of teams and the underlying factors: a mixed-methods study
Source: BMC Fam Pract. 2017 Dec 28;18:111. doi: 10.1186/s12875-017-0685-2 (PMC5745958; doi:10.1186/s12875-017-0685-2)
Supplement: Supplementary file 1 — Questionnaire survey data. The questionnaire used for quantitative data collection on collaboration and relational coordination between primary care professionals. (DOCX 59 kb) [file 12875_2017_685_MOESM1_ESM.docx]

CODE

DATE (DD/MM/JJJJ)

QUESTIONNAIRE

TEAMWORK IN PRIMARY CARE

Dear Sir/Madam,

Before you lies the questionnaire ‘Teamwork in primary care’. This questionnaire is part of a study of the Erasmus University in Rotterdam, Institute of Health Policy and Management on team work in primary care practices. This questionnaire is send to you because you are working as a primary care professional and are involved in the care for elderly people with chronic conditions.

In care for these patients, multiple professionals from different disciplinary backgrounds often have to work together as primary care teams. This study is aimed at gaining more insight in how teamwork is conceptualized in primary care practices and the frequency of contact and communication between professionals from different disciplinary backgrounds.

We kindly ask you to fill in the accompanying questionnaire. This will take around 5-10 minutes. Your input will be of great value for us.

Your answers will remain completely anonymous. The results will only be processed and used by the researchers of the Erasmus University Rotterdam.

**Instructions for filling in the questionnaire**:

X

- Please tick the box with the answer that best suits with your own work situation or circle the right number: 1 2 3 4 5
- If you mistakenly ticked the wrong answer box, please correct your answer by colouring the wrong answer box and ticking the box with your right answer box

X

X

or by putting a cross in the wrong number 1 2 3 4 5 and circle the right number

- There are no wrong or right answers.
- We kindly ask you to fill in all the questions.

**Deadline**

We kindly ask you to send back your filled in questionnaire in the accompanying return envelope within two weeks after receiving this questionnaire.

If you have any questions regarding this questionnaire or would like to receive more information about this study, please feel free to contact the researchers of the Erasmus University:

Kirti Doekhie

Email address: [doekhie@bmg.eur.nl](mailto:doekhie@bmg.eur.nl)

Phone number: ……..................

Thank you very much for your help!

| Background characteristics |
| --- |

1. Date of birth (dd/mm/jjjj): ……………./………………./…………………..
2. Gender

- Male
- Female
- Other, namely………………………………….

1. What is your highest completed eduation level?

- Secondary school
- Secondary vocational
- Bachelor degree
- Master degree
- Other, namely…………………………………………………………..

1. What is your disciplinary background?

- General practitioner
- General practitioners assistant
- Physiotherapist
- Remedial therapist
- Occupational therapist
- Speech therapist
- Primary care psychologist
- Primary care dermatologist
- Dietician
- (District) nurse
- Helping assistant
- Geriatric specialized practice nurse
- Other, namely………………………………………………………………………….

1. How many years have you been active as a professional in your field of expertise?

…………………………. Years

| Primary care team |
| --- |

The next set of questions focus on your teamwork with professionals from other disciplinary backgrounds and the relationship and communication with these professionals.

1. How many team members does your team consist of? If there is a large variation, please give an average:

……………………………………………………. members

1. Which of the following primary care disciplines do you consider part of your team? You may select multiple options.

- General practitioner
- General practitioners assistant
- Physiotherapist
- Remedial therapist
- Occupational therapist
- Speech therapist
- Primary care psychologist
- Primary care dermatologist
- Dietician
- (District) nurse
- Helping assistant
- Geriatric specialized practice nurse
- Other, namely……………………………………………………………………………………

1. Please fill in all the questions for all disciplines that you have selected at question 7.

|  | **A. How frequently do you communicate with each of this disciplines about a patient?** | | | **B. Do these disciplines communicate timely with you?** | | | **C. Do these disciplines communicate accurate with you?** | | | | **D. In case of problems regarding the care for a patient, do these professionals work together with you to fix these problems?** | |
| --- | --- | --- | --- | --- | --- | --- | --- | --- | --- | --- | --- | --- |
|  | **Never Always** | | | **Never Always** | | | **Never Always** | | | | **Never Always** | |
| General practitioner | NA | 1 2 3 4 5 | | N.v.t. | 1 2 3 4 5 | | N.v.t. | | 1 2 3 4 5 | | N.v.t 1 2 3 4 5 | |
| General practitioner assistant | NA | 1 2 3 4 5 | | N.v.t. | 1 2 3 4 5 | | N.v.t. | | 1 2 3 4 5 | | N.v.t 1 2 3 4 5 | |
| Physiotherapist | NA | 1 2 3 4 5 | | N.v.t. | 1 2 3 4 5 | | N.v.t. | | 1 2 3 4 5 | | N.v.t. 1 2 3 4 5 | |
| Remedial therapist | NA | 1 2 3 4 5 | | N.v.t. | 1 2 3 4 5 | | N.v.t. | | 1 2 3 4 5 | | N.v.t 1 2 3 4 5 | |
| Occupational therapist | NA | 1 2 3 4 5 | | N.v.t. | 1 2 3 4 5 | | N.v.t. | | 1 2 3 4 5 | | N.v.t 1 2 3 4 5 | |
| Speech therapist | NA | 1 2 3 4 5 | | N.v.t. | 1 2 3 4 5 | | N.v.t. | | 1 2 3 4 5 | | N.v.t 1 2 3 4 5 | |
| Primary care psychologist | NA | 1 2 3 4 5 | | N.v.t. | 1 2 3 4 5 | | N.v.t. | | 1 2 3 4 5 | | N.v.t 1 2 3 4 5 | |
| Primary care dermatologist | NA | 1 2 3 4 5 | | N.v.t. | 1 2 3 4 5 | | N.v.t. | | 1 2 3 4 5 | | N.v.t 1 2 3 4 5 | |
| Dietician | NA | 1 2 3 4 5 | | N.v.t. | 1 2 3 4 5 | | N.v.t. | | 1 2 3 4 5 | | N.v.t 1 2 3 4 5 | |
| (District) nurse | NA | 1 2 3 4 5 | | N.v.t. | 1 2 3 4 5 | | N.v.t. | | 1 2 3 4 5 | | N.v.t 1 2 3 4 5 | |
| Helping assistant | NA | 1 2 3 4 5 | | N.v.t. | 1 2 3 4 5 | | N.v.t. | | 1 2 3 4 5 | | N.v.t 1 2 3 4 5 | |
| Geriatric specialized practice nurse | NA | 1 2 3 4 5 | | N.v.t. | 1 2 3 4 5 | | N.v.t. | | 1 2 3 4 5 | | N.v.t 1 2 3 4 5 | |
| Other, namely………………………………. | NA | 1 2 3 4 5 | | N.v.t. | 1 2 3 4 5 | | N.v.t. | | 1 2 3 4 5 | | N.v.t 1 2 3 4 5 | |
|  |  |  | |  |  | |  | |  | |  | |
|  | **E. To what degree do these disciplines understand your role in the team?** | | | **F. To what degree do these disciplines respect you?** | | | | **G. To what degree do these disciplines share your goals for the care of your patients?** | | | |  |
|  | **Never Always** | | | **Never Always** | | | | **Never Always** | | | |  |
| General practitioner | NA | | 1 2 3 4 5 | NA | | 1 2 3 4 5 | | NA | | 1 2 3 4 5 | |  |
| General practitioner assistant | NA | | 1 2 3 4 5 | NA | | 1 2 3 4 5 | | NA | | 1 2 3 4 5 | |  |
| Physiotherapist | NA | | 1 2 3 4 5 | NA | | 1 2 3 4 5 | | NA | | 1 2 3 4 5 | |  |
| Remedial therapist | NA | | 1 2 3 4 5 | NA | | 1 2 3 4 5 | | NA | | 1 2 3 4 5 | |  |
| Occupational therapist | NA | | 1 2 3 4 5 | NA | | 1 2 3 4 5 | | NA | | 1 2 3 4 5 | |  |
| Speech therapist | NA | | 1 2 3 4 5 | NA | | 1 2 3 4 5 | | NA | | 1 2 3 4 5 | |  |
| Primary care psychologist | NA | | 1 2 3 4 5 | NA | | 1 2 3 4 5 | | NA | | 1 2 3 4 5 | |  |
| Primary care dermatologist | NA | | 1 2 3 4 5 | NA | | 1 2 3 4 5 | | NA | | 1 2 3 4 5 | |  |
| Dietician | NA | | 1 2 3 4 5 | NA | | 1 2 3 4 5 | | NA | | 1 2 3 4 5 | |  |
| (District) nurse | NA | | 1 2 3 4 5 | NA | | 1 2 3 4 5 | | NA | | 1 2 3 4 5 | |  |
| Helping assistant | NA | | 1 2 3 4 5 | NA | | 1 2 3 4 5 | | NA | | 1 2 3 4 5 | |  |
| Geriatric specialized practice nurse | NA | | 1 2 3 4 5 | NA | | 1 2 3 4 5 | | NA | | 1 2 3 4 5 | |  |
| Other namely…………………. | NA | | 1 2 3 4 5 | NA | | 1 2 3 4 5 | | NA | | 1 2 3 4 5 | |  |

1. If you have any additional remarks or feedback regarding the questionnaire, please feel free to write these down below in the text box.

**You have reached the end of this questionnaire. We kindly ask you to check whether you have filled in all the questions. Thank you so much for your time and effort.**
