# Supplementary material for: Who is on the primary care team? Professionals’ perceptions of the conceptualization of teams and the underlying factors: a mixed-methods study
Source: BMC Fam Pract. 2017 Dec 28;18:111. doi: 10.1186/s12875-017-0685-2 (PMC5745958; doi:10.1186/s12875-017-0685-2)
Supplement: Supplementary file 2 — Interview guide. The interview guide used for qualitative data collection on collaboration and teams. (DOCX 16 kb) [file 12875_2017_685_MOESM2_ESM.docx]

ADDITIONAL FILE 2

SEMI –STRUCTED INTERVIEW GUIDE

**Part A: Introduction and consent**

1. Express thank you for time and effort to do the interview
2. Short introduction of the researcher’s background and goal of the study
3. Discuss informed consent from participants:

- Ask whether participants still consent to the interview (verbal consent)
- Ask consent to the use of the transcript and quotes for the purpose of this study (verbal consent)
- Clearly state that all results will remain anonymous

1. Discuss verbal consent for recording of the interviews:

- clearly state again that all results will remain anonymous
- Ask if participants consent to recording of the interview

1. Inform that we are particularly interested in example cases.

**Part B: description of the participant**

| Question | Follow up question(s) | Topics to discuss |
| --- | --- | --- |
| 1. Could you share some information about your professional background? |  | Disciplinary background, years active in the field, type of organisation |
| 1. How are you involved in the care for elderly patients with chronic conditions? |  | Involvement in the for elderly patients with chronic conditions |

**Part C: collaboration professionals with other disciplinary backgrounds**

| Question | Follow up question(s) | Topics to discuss |
| --- | --- | --- |
| 1. Do you collaborate with professionals from other disciplinary backgrounds in the care for elderly patients with chronic conditions? | If answer is yes:   1. Could you share something about what this collaboration entails? | Type of contact between professionals from different disciplinary backgrounds |
| 1. How would you describe your own role in these collaborations? |  | Leadership, division of tasks |
| 1. What are your feelings on how on well or not well the collaboration with other professionals currently is going? | 1. What are positive elements of this collaboration? 2. On which elements would you like to see improvement or change? | Opinion on the quality of collaborations between professionals from different disciplinary backgrounds |
| 1. What is important to you when collaborating with other disciplines? |  | Crucial elements of collaborations |
| 1. How do you communicate with these professionals regarding a patient’s medical condition and care goals? |  | Type and frequency of contact and communication |

**Part D: primary care teams**

| 1. Often, collaborations are labelled as primary care teams. Do you feel to be part of a team? | 1. Why (not)? | Sense of belonging to a team |
| --- | --- | --- |
| 1. How would you define a primary care team? |  | Definition and conceptualization of a team |
| 1. Do you find it important to work as a team with other professionals? |  | Added value of teamwork, willingness to work as a team |
| 15. What elements of teamwork could make you feel more like a member of a team? |  |  |
