# Supplementary material for: Who is on the primary care team? Professionals’ perceptions of the conceptualization of teams and the underlying factors: a mixed-methods study
Source: BMC Fam Pract. 2017 Dec 28;18:111. doi: 10.1186/s12875-017-0685-2 (PMC5745958; doi:10.1186/s12875-017-0685-2)
Supplement: Supplementary file 3 — Team size, diversity and part of the team. The table indicates the average team size and diversity and which primary care disciplines are considered as part of the team by professionals from other disciplinary backgrounds. (DOCX 14 kb) [file 12875_2017_685_MOESM3_ESM.docx]

|  | | **team size** | **diversity** | **considered part of the team (% yes)** | | | | | | | | | | |
| --- | --- | --- | --- | --- | --- | --- | --- | --- | --- | --- | --- | --- | --- | --- |
| **In eyes of:** | | mean | mean (SD) | 1 | 2 | 3 | 4 | 5 | 6 | 7 | 8 | 9 | 10 | 11 |
|  | Total respondents group  (n=159) | 10,28 | 0,45 (0,31) | 51,9 | 41,7 | 40,4 | 39,1 | 28,2 | 25,6 | 19,9 | 17,9 | 14,7 | 10,3 | 4,5 |
| 1 | Physiotherapist  (n=36) | 9,18 | 0,44 (0,28) | 94,4 | 25 | 27,8 | 19,4 | 11,1 | 41,7 | 30,6 | 38,9 | 30,6 | 8,3 | 0 |
| 2 | (District) Nurse  (n=19) | 8,26 | 0,60 (0,21) | 47,4 | 100 | 42,1 | 89,5 | 21,1 | 10,5 | 21,1 | 0 | 0 | 0 | 0 |
| 3 | General Practitioner  (n=9) | 10 | 0,64 (0,11) | 11,1 | 0 | 88,9 | 0 | 88,9 | 11,1 | 0 | 33,3 | 0 | 0 | 0 |
| 4 | Helping Assistant  (n=31) | 10,19 | 0,58 (0,28) | 38,7 | 87,1 | 45,2 | 96,8 | 29 | 19,4 | 32,3 | 6,5 | 12,9 | 12,9 | 6,5 |
| 5 | Geriatric Specialized Practice Nurse  (n=5) | 10,5 | 0,75 (0,10) | 20 | 20 | 80 | 20 | 80 | 60 | 0 | 0 | 0 | 0 | 0 |
| 6 | Dietician  (n=5) | 9,6 | 0,45 (0,14) | 40 | 0 | 20 | 0 | 20 | 100 | 40 | 40 | 40 | 0 | 0 |
| 7 | Occupational Therapist  (n=2) | 15 | 0,79 (0,09) | 100 | 100 | 50 | 100 | 0 | 100 | 100 | 100 | 0 | 0 | 0 |
| 8 | Primary Care Psychologist  (n=2) | 7 | 0,36 (0,12) | 100 | 50 | 0 | 0 | 0 | 0 | 0 | 100 | 0 | 0 | 0 |
| 9 | Speech Therapist  (n=2) | 13,5 | 0,74 (0,13) | 100 | 100 | 28,6 | 100 | 0 | 100 | 100 | 100 | 100 | 50 | 0 |
| 10 | Remedial Therapist  (n=22) | 9,81 | 0,10 (0,20) | 77,3 | 100 | 9,1 | 0 | 4,5 | 9,1 | 0 | 100 | 4,5 | 36,4 | 0 |
| 11 | Primary Care Dermatologist  (n=5) | 15 | 0 (0) | 0 | 0 | 0 | 0 | 0 | 0 | 0 | 0 | 0 | 0 | 83,3 |

Additional file 3. Team size, diversity and part of the team
