# Supplementary material for: Who is on the primary care team? Professionals’ perceptions of the conceptualization of teams and the underlying factors: a mixed-methods study
Source: BMC Fam Pract. 2017 Dec 28;18:111. doi: 10.1186/s12875-017-0685-2 (PMC5745958; doi:10.1186/s12875-017-0685-2)
Supplement: Supplementary file 4 — Mutual degrees of relational coordination, subdivided into communication and relationship dimensions. The table indicates the degrees of relational coordination between primary care professionals from different disciplinary backgrounds as perceived by the professionals themselves. (DOCX 14 kb) [file 12875_2017_685_MOESM4_ESM.docx]

| **With discipline:**  **Relational coordination in eyes of:** | Physiotherapist | (District) Nurse | General Practitioner | Helping Assistant | Geriatric Specialized Practice nurse | Dietician | Remedial Therapist | Primary Care Dermatologist |
| --- | --- | --- | --- | --- | --- | --- | --- | --- |
| Physiotherapist | 3.68/ 3.94 | 2.74/ 2.96 | 2.95/ 3.64 | 2.81/ 3.03 | 2.04/ 2.50 | 2.46/ 2.74 | 1.83/ 1.96 | 1.19/ 1.26 |
| (District) Nurse | 2.85/ 3.08 | 4.54/ 4.61 | 3.43/ 3.64 | 4.54/ 4.49 | 2.88/ 3.27 | 2.52/ 2.61 | 1.45/ 1.47 | 1.41/ 1.72 |
| General Practitioner | 2.37/ 2.76 | 2.40/ 2.86 | 2.44/ 2.48 | 2.02/ 2.57 | 3.83/ 4.10 | 2.35/ 2.14 | 1.80/ 2.76 | 1.00/ 1.62 |
| Helping Assistant | 2.10/ 2.59 | 4.74/ 4.78 | 3.07/ 3.63 | 4.72/ 4.80 | 2.55/ 3.00 | 1.71/ 1.74 | 1.45/ 1.63 | 1.31/ 1.39 |
| Geriatric Specialized Practice nurse | 2.56/ 2.67 | 3.06/ 2.83 | 4.65/ 4.73 | 2.81/ 2.83 | 4.60/ 4.67 | 4.06/ 4.67 | 2.00/ 2.00 | 2.00/ 2.00 |
| Dietician | 1.60/ 2.20 | 1.00/ 1.00 | 3.50/ 3.87 | 1.25/ 1.27 | 2.78/ 2.87 | 2.63/ 2.53 | 1.00/ 1.00 | 1.00/ 1.00 |
| Remedial Therapist | 3.10/ 3.49 | 1.23/ 1.32 | 2.60/ 3.18 | 1.25/ 1.35 | 1.79/ 2.18 | 1.88/ 2.30 | 2.88/ 3.21 | 1.00/ 1.20 |
| Primary Care Dermatologist | 1.00/ 1.61 | 1.17/ 1.50 | 2.46/ 3.00 | 1.17/ 1.50 | 2.04/ 2.61 | 1.00/ 1.33 | 1.00/ 1.33 | 3.63/ 3.89 |
| General Practitioner Assistant | 1.55/ 2.09 | 1.75/ 1.95 | 4.36/ 4.75 | 1.70/ 2.11 | 3.65/ 4.51 | 1.67/ 2.45 | 1.14/ 1.36 | 1.00/ 1.39 |
| Mean score communication dimensions/mean score relationship dimensions  Groups lower than n=5 are eliminated | | | | | | | | |

Additional file 4

Mutual degrees of relational coordination, subdivided into communication and relationship dimensions
