# Supplementary material for: Who is on the primary care team? Professionals’ perceptions of the conceptualization of teams and the underlying factors: a mixed-methods study
Source: BMC Fam Pract. 2017 Dec 28;18:111. doi: 10.1186/s12875-017-0685-2 (PMC5745958; doi:10.1186/s12875-017-0685-2)
Supplement: Supplementary file 5 — Descriptives and correlations between perceived as part of the team and the degree of relational coordination. The table indicates the correlation between the perceptions of all participants on the team membership of professionals from a specific disciplinary background in the team and the perceived degrees of relational coordination with these professionals. (DOCX 13 kb) [file 12875_2017_685_MOESM5_ESM.docx]

|  | **Perceived as part of the team by all participants** | **Perceived degree of relational coordination by all participants** | | | |
| --- | --- | --- | --- | --- | --- |
|  |  | **Communication dimensions** | | **Relationship**  **dimensions** | |
| **Discipline** | % | Mean | Correlation (r) | Mean | Correlation (r) |
| General Practitioner | 40.9 | 3.14 | .35* | 3.65 | .17** |
| Physiotherapist | 54.4 | 2.71 | .55* | 3.10 | .49* |
| (District) Nurse | 42.3 | 2.96 | .76* | 3.09 | .67* |
| Helping Assistant | 39.6 | 2.94 | .76* | 3.10 | .68* |
| Practice Nurse*** | 29.5 | 2.58 | .51* | 2.99 | .47* |
| Dietician | 26.8 | 2.17 | .53* | 2.39 | .43* |
| Remedial Therapist | 10.7 | 1.73 | .55* | 1.95 | .45* |
| Occupational Therapist | 20.8 | 2.05 | .42* | 2.37 | .39* |
| PC Dermatologist | 4.7 | 1.30 | .50* | 1.47 | .48* |
| Pharmacist | 16.8 | 2.00 | .32* | 2.36 | .25* |
| PC Psychologist | 18.8 | 1.70 | .65* | 1.93 | .56* |
| Speech Therapist | 15.5 | 1.65 | .54* | 1.87 | .49* |
| * Significant at .01 level  ** Significant at .05 level  *** The category ‘Practice Nurse’ refers to the Geriatric Specialized Practice Nurses | | | | | |

Additional file 5.

Descriptives and correlations between perceived as part of the team and the degree of relational coordination
